# FAMILY-MEMBER LOSS AS A CUMULATIVE BURDEN FOR HEALTH AMONG BLACK AND WHITE OLDER ADULTS

**DOI:** 10.1093/geroni/igz038.2248

**Published:** 2019-11-08

**Authors:** Rachel Donnelly

**Affiliations:** Vanderbilt University, Nashville, Tennessee, United States

## Abstract

The health consequences of multiple family member deaths across the life course has received less attention in the bereavement literature. Moreover, recent research shows that black Americans are more likely than white Americans to lose multiple family members. I analyze longitudinal data from the Health and Retirement Study (1992-2014) to assess how multiple family member losses across the life course are associated with declines in health among older adults. Findings suggest that multiple family losses prior to midlife are associated with a number of indicators of poor health (e.g., functional limitations, cardiometabolic health) and steeper declines in health as individuals age. Losses after midlife additionally undermine health declines for older adults. Thus, family member loss functions as a cumulative burden of stress across the life course that erodes health in mid- and later-life. Family loss disproportionately burdens black Americans and serves as a unique source of disadvantage for black families.

